# Research on Adhesion Performance of Track Monomer with Bionic Structure

**DOI:** 10.3390/biomimetics10040250

**Published:** 2025-04-18

**Authors:** Sanling Fu, Xiahua Cui, Le Yang, Xinyue Wang, Zhijun Guo, Fu Zhang

**Affiliations:** 1 Longmen Laboratory, Luoyang 471000, China; 9903401@haust.edu.cn; 2 College of Physical Engineering, Henan University of Science and Technology, Luoyang 471023, China; 3 College of Biological and Agricultural Engineering, Jilin University, Changchun 130025, China; cuixh23@mails.jlu.edu.cn (X.C.); xyw24@mails.jlu.edu.cn (X.W.); 4 College of Agricultural Equipment Engineering, Henan University of Science and Technology, Luoyang 471003, China; yangle@stu.haust.edu.cn; 5 College of Vehicle & Transportation Engineering, Henan University of Science and Technology, Luoyang 471003, China; gzhj1970@haust.edu.cn

**Keywords:** goat spine, spinal space curve, track monomer, bionic structure, adhesion performance

## Abstract

Goats can walk freely and flexibly in complex environments such as concave and convex or soft ground. And their flexible spine has functions such as adjusting balance and providing auxiliary power during movement, while the limbs only have support functions. The spine has an adjustable and decisive role in the pressure on the sole of the hoof of the goat. Therefore, the goat spine was taken as the bionic prototype, the three-dimensional force distribution of the goat body space was analyzed, and the optimal spinal space curve was explored, combined with the goat gait cycle. Based on the study of spinal curve arrangement and placement, the spinal curve was stretched along the grouser length direction. The soil contact surface structure of the track monomer was constructed based on functional simulation. And the bionic structure of the track monomer with superior adhesion performance was explored. The results of simulation analysis and soil tank test both showed that the attachment performance of bionic structure was better than that of an ordinary structure. It showed that adding bionic curves to the contact surface of the track monomer could significantly improve the adhesion performance, and the bionic structure with a single bionic curve arranged on the complete contact surface of the track monomer had the best adhesion performance. Moreover, the adhesion of the optimal track monomer bionic structure was increased by 19.22 N compared with an ordinary structure in the soil tank test, which verified the superiority of the track monomer bionic structure design. It provides a new method and a new idea for improving the adhesion performance of tracked vehicle in hilly areas.

## 1. Introduction

Hills and mountains are important production bases for grain, oil, sugar, and characteristic agricultural products in China. Crawler agricultural machinery commonly used in this area has the characteristics of a large contact area and strong ground adaptability. However, when it travels in soft soil, the adhesion provided by the soil to the track is insufficient, which is easy to lead to skidding and poor adhesion performance. Its passing performance is seriously affected. Therefore, improving track adhesion performance is an important part of the current research and development of agricultural equipment in hilly and mountainous areas [1,2,3,4].

The track monomer is a key component of soil contact, and the adhesion performance of track agricultural equipment depends largely on its structural parameters [5]. Zhang et al. [6] studied the influence of structural parameters such as track width, support section length and spike height on the traction adhesion performance of micro-crawler tractors in combination with orthogonal tests and found that spike height had the greatest impact on adhesion and could significantly improve vehicle adhesion performance. Yang et al. [7] analyzed the soil thrust of a track plate based on the soil moisture content and shear rate, studied the influence of a grouser draft angle and splay angle on soil thrust and modified the soil thrust formula of track plates in combination with the traction test of track plates. Li et al. [8] took π-shaped, T-shaped and V-shaped tracks as research objects and studied the influence of track structure parameters of an unmanned underwater crawler bulldozer on traction performance by changing structural parameters such as length, width and height, so as to provide theoretical reference for optimizing track structure and improving the traction performance of an unmanned underwater crawler bulldozer. Fu et al. [9] took adhesion and clay quality as response indexes and combined them with the mathematical model of structural parameter–response indexes to study the optimal combination of structural parameters such as the height, thickness and angle of the shoe. They found that the optimal track parameter combination was 20 mm in height, 6.34 mm in width and 40.45° in the angle of the shoe. The research on adhesion performance of the track monomer mainly focused on the influence of its parameters on performance under different environmental conditions and the optimization of pattern structure parameters. However, there are few reports on regulating the interaction between track monomers and soil through the structural design of the soil contact surface of the track monomer matrix, so as to improve its adhesion performance.

The spinal structure of mammals provides auxiliary power during movement, while the limbs only provide support. Through spinal movement, goats can dynamically adjust the trunk-pitching posture and affect the hoof pressure distribution, so that they can display a variety of dexterous movement posture and show strong athletic ability. Rifkin et al. [10] studied the vertical reaction of sheep walking on flat ground and found that the front hoof bore more force than the back hoof and that the vertical reaction distribution law of sheep was similar to that of horses and dogs. Xiangyu Liu et al. [11] studied the changes in vertical reaction of the limbs of sheep with different gradients. It was found that the vertical reaction of the hind limbs was greater and increased with the increase in slope, and the difference index of the left and right sides of the limbs had similar changes. Plantar pressure distribution has a significant impact on the body’s locomotion ability. Qi Zhang [12] studied the dynamic morphological characteristics of a goat’s foot based on the magnitude, direction and area of hoof pressure during movement. And the magnitude and distribution characteristics of hoof pressure were analyzed in different movement modes. It was found that the peak value of full hoof pressure increased from 57.5% of body weight to 92.5%, while the pressure of the shoe ball increased from 52% to 78%. At present, the research on the biological characteristics of the spine mainly focuses on the study of the structural characteristics and mechanical properties of the anatomical spine or analyzing the spinal motion characteristics of animals in different motion states using high-speed photography. However, there are few reports on the application of dynamic characteristics of the spine to the bionic structure of track monomers based on functional simulation. It will make the pressure distribution of the track monomer contact surface behave like the pressure adjustment of the goat’s foot when the soil interacts with the spinal track-like monomer substrate.

To evaluate the adhesion performance of tracked plates under clay conditions, Fu et al. adopted adhesion force as the evaluation index and examined the effects of height, thickness, and opening angle of the tracked spur structure on adhesion performance under black clay conditions [13,14]. Li et al. [15]. designed a set of bionic grousers inspired by buffalo hooves, to ensure stable movement of these vehicles across deep-sea sediment. The simulation results of the bionic grouser are compared with those of conventional straight grousers, highlighting the advantages of the bionic design in enhancing traction. This analysis provides valuable insights for the optimization of grouser designs for deep-sea mining vehicles.

Therefore, the adhesion performance of the goat matrix was quantitatively described, combined with the spinal space curve equation, and the adhesion coefficient was taken as the evaluation index to obtain the optimal spinal space curve with the bionic prototype of the spine of the goat. Based on the principle of bionics, the dynamic characteristics of the spine are applied to design the bionic structure of the track monomer. The results of simulation analysis and soil tank test both showed that the attachment performance of bionic structure was better than that of the ordinary structure. It showed that adding bionic curves to the contact surface of the track monomer could significantly improve the adhesion performance, and the bionic structure with a single bionic curve arranged on the complete contact surface of the track monomer had the best adhesion performance. Moreover, the adhesion of the optimal track monomer bionic structure was increased by 19.22 N compared with an ordinary structure in the soil tank test, which verified the superiority of the track monomer bionic structure design. It provides a new method and a new idea for improving the adhesion performance of tracked vehicles in hilly areas.

## 2. Materials and Methods

### 2.1. Analysis of Interaction Between Track Monomer and Soil

**Figure 1:**
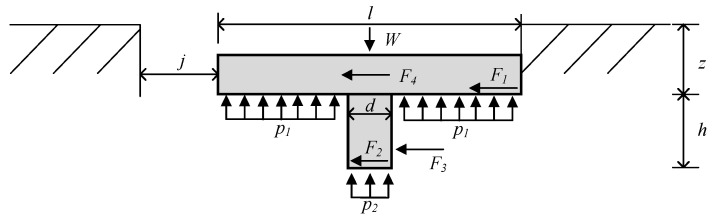
Force analysis of straight track monomer.

Track monomer—ground adhesion model is an important basis for the research of track monomer adhesion performance. The important factors affecting track monomer adhesion can be obtained by analyzing the adhesion model. Figure 1 shows the stress state of conventional one-frame track monomer when shearing soil.

Where *F*_1_ represents the horizontal force on the bottom of the track monomer matrix, *F*_2_ represents the horizontal force on the bottom of the track grouser, *F*_3_ represents the horizontal force perpendicular to the piercing shear plane, *F*_4_ represents the horizontal force on both ends of the track monomer and the spine, respectively, *p*_1_ represents the soil stress on the bottom of the substrate when the track monomer sinks to a certain depth under the action of load, *p*_2_ represents the soil stress on the bottom of the track unit when it sinks to a certain depth under the action of load, *z* is amount of soil subsidence, h is track grouser height, d is the thickness of the track grouser, l is the length of the track monomer, j is the travel distance of the track monomer, and W is the vertical load on the track monomer. Then, the maximum thrust generated by the track monomer shearing the soil is shown in Formula (1),

(1) $$F_{max}=F_{1}+F_{2}+F_{3}+F_{4}$$

Known from Coulomb soil breaking Formula (2),

(2) $$\tau_{f}=c+\sigma \tan\varphi$$

where $\tau_{f}$ is soil shear strength, *c* is soil cohesive strength, φ is the angle of internal friction and σ is vertical stress.

A study conducted by Congbin Yang showed that shear rate and soil water content are important factors affecting soil shear strength [16]. After water content and shear rate were taken into account, the formula of shear strength was modified as Formula (3)

(3) $$\left\{\begin{array}{c} \tau_{f}=\eta \left(v\right)\left[c\left(w\right)+\sigma \tan\varphi_{u}\left(w\right)\right]\\ c\left(w\right)=c_{0}+\lambda_{c}w\\ \varphi_{u}\left(w\right)=\varphi_{0}{\left(w+1\right)}^{n_{\varphi }}\\ \eta \left(v\right)=\frac{\tau }{\tau_{0}}=\frac{\eta_{max}}{v+a} \end{array}\right.$$

where w is soil water content, $c_{0}$ represents soil cohesion coefficient when water content is 0, $\lambda_{c}$ is the slope of soil cohesion strength curve, $\varphi_{0}$ is soil internal friction angle when water content is 0, $n_{\varphi }$ is fitting coefficient of internal friction angle curve ($-1<n_{\varphi }<0$), η is amplification factor, $\eta_{max}$ is the maximum critical value of the amplification factor, *v* is soil shear rate, *a* is the value of the test velocity in relation to soil, and $\tau_{0}$ represents soil shear strength at low shear rate.

Known from Bekker sinking Formula (4) [17],

(4) $$\left\{\begin{array}{c} p_{1}=\left(\frac{k_{c}}{b}+k_{\varphi }\right)z^{n}\\ p_{2}=\left(\frac{k_{c}}{b}+k_{\varphi }\right){\left(z+h\right)}^{n} \end{array}\right.$$

where $k_{c}$ is soil adhesion deformation modulus, $k_{\varphi }$ is soil friction deformation modulus, d represents track width and n represents soil deformation index, dimensionless.

According to Newton’s second law, the bottom stresses of the track monomer matrix and the grouser are satisfied, shown in Formula (5):

(5) $$p_{1}\left(l-d\right)b+p_{2}db=W$$

Forces *F*_1_ and *F*_2_ can be solved by integration,

(6) $$F=b\int_{0}^{1}\tau \left(x\right)dx$$

where $\tau \left(x\right)$ represents mathematical relationship between soil shear stress and displacement.

The maximum horizontal force *F*_1_ on the bottom of the track monomer substrate and *F*_2_ on the bottom of the grouser are, respectively,

(7) $$\begin{array}{l} F_{1}=b\left(l-d\right)\eta \left(v\right)\left[c\left(w\right)+\left(\frac{k_{c}}{b}+k_{\varphi }\right)z^{n}\tan\varphi_{u}\left(w\right)\right]\\ =b\left(l-d\right)\eta \left(v\right)\left[c\left(w\right)+p_{1}\tan\varphi_{u}\left(w\right)\right] \end{array}$$

(8) $$\begin{array}{l} F_{2}=bd\eta \left(v\right)\left[c\left(w\right)+\left(\frac{k_{c}}{b}+k_{\varphi }\right){\left(z+h\right)}^{n}\tan\varphi_{u}\left(w\right)\right]\\ =bd\eta \left(v\right)\left[c\left(w\right)+p_{2}\tan\varphi_{u}\left(w\right)\right] \end{array}$$

Therefore, the soil stress is on the bottom of the track monomer matrix and the bottom of the grouser surface. That is, the interaction between track monomer and soil is the key factor affecting the maximum thrust of track monomer when shearing soil.

### 2.2. Bionic Structure Design of Track Monomer

The previous study [18] found that both the thoracic and lumbar vertebra movements of goats showed coronal side to side swing. And the general equation of spinal space fitting curve could be expressed as

(9) $$\left\{ \begin{array}{c} y=f(x)=ax^{3}+b_{1}x^{2}+cx+d\\ z=f(x)=ex^{2}+fx+g \end{array}\right.$$

**Figure 2:**
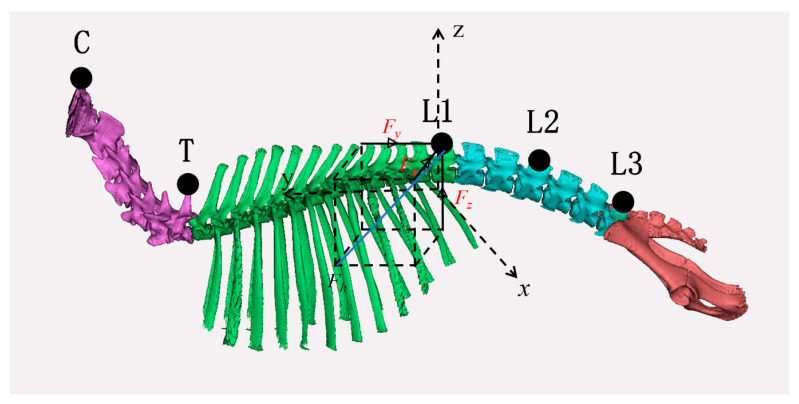
Diagram of three-dimensional force distribution in space of goat [19].

On the basis of the above, a three-dimensional spatial mechanical analysis of the goat body was conducted as a whole, and the three-dimensional spatial force distribution was shown in Figure 2. Where $F_{x}$ represents spatial lateral force, N, $F_{y}$ represents spatial forward driving force, N, $F_{z}$ represents vertical ground reaction force, N, and $F_{h}$ represents three-dimensional resultant force of space, N.

Because the three-dimensional force plate was subjected to friction during goat walking, noise was generated and data analysis was affected. Six data-smoothing methods of moving, lowess, loess, sgolay, rlowess and rloess, were adopted, respectively, and the signal-to-noise ratio (SNR), root-mean-square error (RMSE) and coefficient of determination (*R*^2^) were taken as evaluation indexes. The optimal data-smoothing method was selected as the final pressure de-noising method.

The de-noising results of space lateral force, space forward driving force and vertical ground reaction force under the slope of 10° are shown in Table 1. The larger the SNR value, the smaller the noise, and the higher the data reliability. The smaller the RMSE value, the smaller the deviation between the de-noised data and the original data. The larger the R^2^, the higher the fitting degree between the de-noised data and the original data.

**Figure 3:**
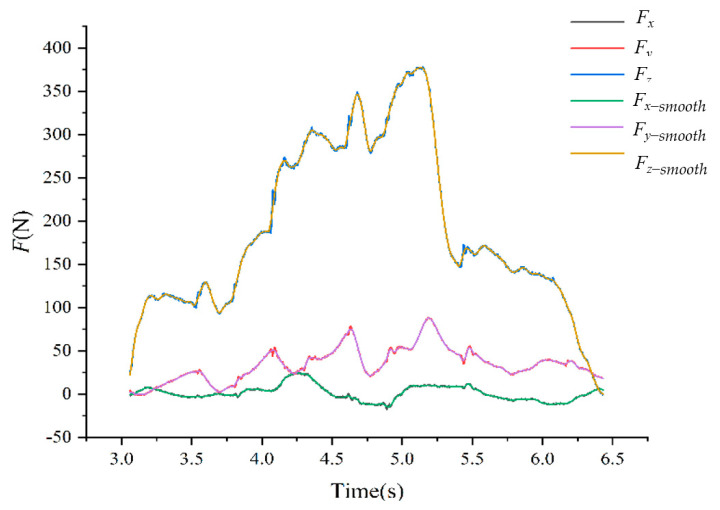
The comparison of the force values before and after the denoising method of loess.

The comprehensive analysis showed that the SNR and R^2^ of the loess smoothing method were the largest, while the RMSE was the smallest. So it had the best de-noising effect. The comparison of the force values before and after the denoising method of loess is shown in Figure 3.

The analysis of Table 1 shows that the loess method has the best denoising effect, and Figure 3 shows the comparison of the force values before and after denoising of this method, in which the spatial three-dimensional force during the movement of the goat was mainly manifested as the spatial forward driving force *F_y_* and the vertical ground reaction force *F_z_*. The change in the attachment coefficient during the movement of the goat was minimized by the *F_x-smooth_.* Formula (10) for calculating adhesion coefficient is as follows:

(10) $$\mu =\frac{F_{y}}{F_{z}}$$

where μ represents adhesion coefficient.

In Formula (10), the instantaneous state force values of goat movement were taken as *F_y_* and *F_z_*. In order to accurately analyze the effect of goat spine motion on its hoof attachment coefficient, the force value of 0.01 s sampling interval was retained. Data were analyzed from 3.90 s (left front leg, right front leg and right hind leg supported by the three-dimensional pressure plate, the first moment of left hind leg swung) to 5.17 s (left front leg, left hind leg and right hind leg supported by the three-dimensional pressure plate, the last moment of right front leg swung).

**Figure 4:**
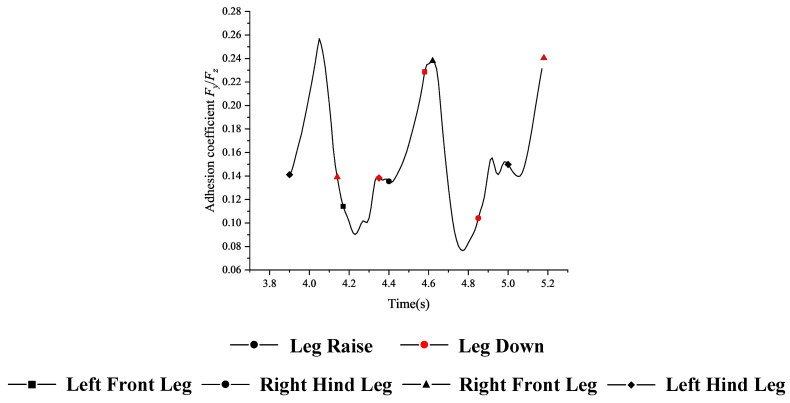
Variation in adhesion coefficient.

As can be seen from Figure 4, there were multiple peaks in the attachment coefficient. The attachment coefficient reached its maximum at the moment when the right hind leg swung, the left hind leg and the left front leg supported, and the right front leg started to swing. The spatial spinal curve equation for this moment was

(11) $$\left\{\begin{array}{c} y=f\left(x\right)=-1.48e^{-6}x^{3}+0.003x^{2}-1.86x+536.52\\ y=f\left(x\right)=-14.77e^{-5}x^{2}+0.27x+415.68 \end{array}\right.$$

**Figure 5:**
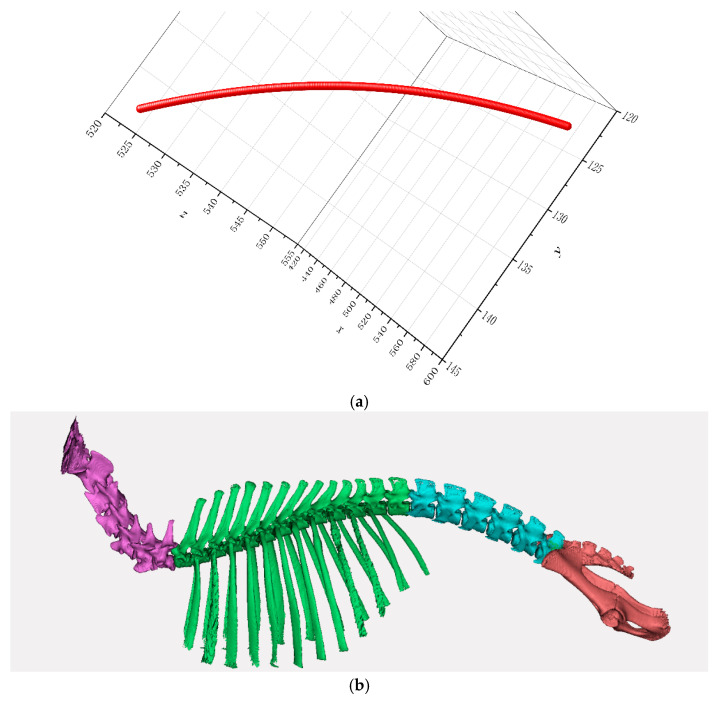
(**a**) Spatial of the spine with maximum adhesion coefficient. (**b**) A 3D model of the spine after each structure shows differentiation.

Spatial of the spine with maximum adhesion coefficient is shown in Figure 5.

The optimal spinal coronal plane curve equation was selected and imported into SolidWorks software to obtain the bionic curve combined with the conclusion that the spinal coronal plane swing had the most significant influence on its movement. It was regarded as a rigid structure with the high stability of the thoracic vertebra, so the thoracic vertebra part was discarded, and the lumbar vertebra curve was retained to design a monomer bionic structure. The theoretical equation of monomer bionic structure was obtained with the value range of *x*,

(12) $$f(x)=-1.48e^{-6}x^{3}+0.003x^{2}-1.86x+536.52,\;431.81\leq x\leq 580.81\;\mathrm{mm}$$

**Figure 6:**
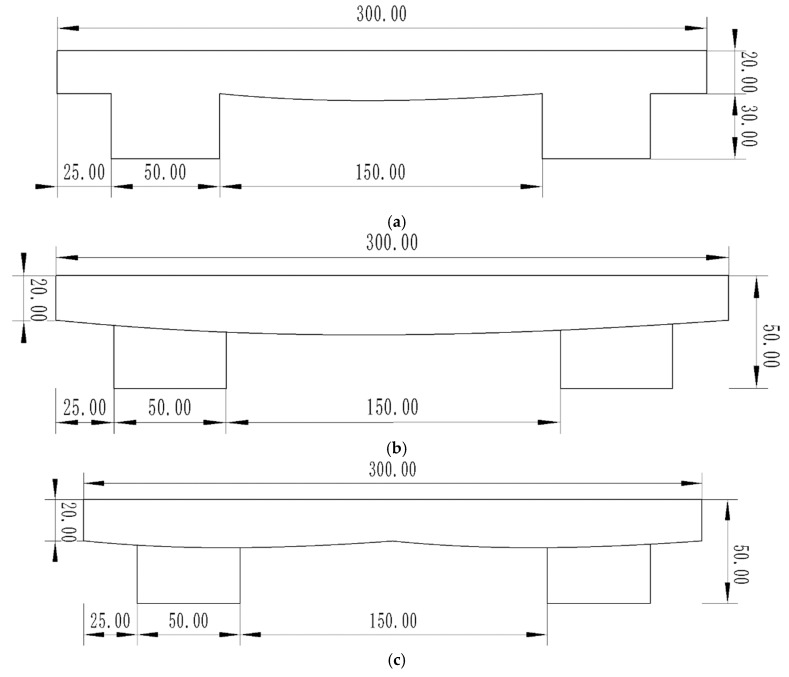

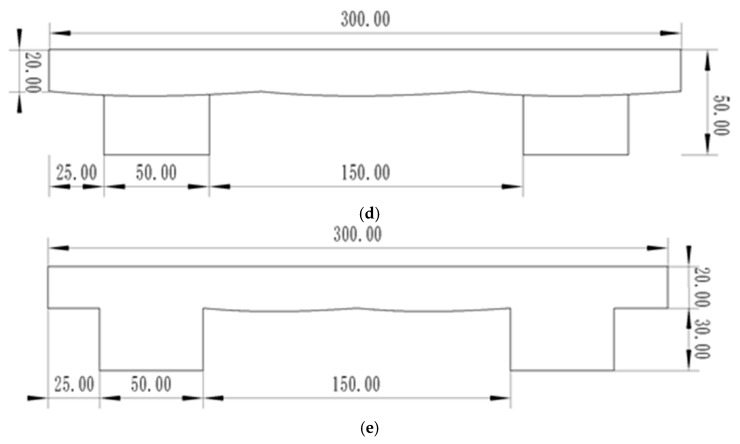
Schematic diagram of track monomer bionic structures: (**a**) Bionic structure A of the track monomer. (**b**) Bionic structure C of the track monomer. (**c**) Bionic structure D of the track monomer. (**d**) Bionic structure E of the track monomer. (**e**) Bionic structure F of the track monomer.

The conventional single-frame track monomer was taken as the research object, and the theoretical curve was arranged on the ground contact surface of the base part of the track monomer (between the two grousers). The theoretical curve was stretched along the length of the grouser, and the bionic structure was constructed to realize the interaction between the contact surface of the track monomer and the soil. The structure parameters of the base part of track monomer were length × width × height = 300 mm × 300 mm × 20 mm. In order to explore the influence of curve arrangement and layout on the adhesion performance of the track monomer, structures A, C, D, E, and F (unit: mm) were designed, respectively, with the same structural parameters, as shown in Figure 6.

### 2.3. Construction of Track Monomer-Soil Interaction Simulation Model

(1) Material intrinsic parameters

The track monomer samples involved were all made of rubber, and the intrinsic parameters of rubber were obtained by referring to relevant references [20]: density of 960 g/cm^3^, Poisson’s ratio of 0.45, and elastic modulus of 3448 Pa.

The soil was selected from the soil tank test bench. Before sampling, the soil was evenly loosened with a soil-loosening tool. Test samples were obtained from 5 places in the soil tank test bench with a ring knife according to the five-point sampling method.

(1.1) Soil moisture content

Soil moisture content is an important parameter affecting the adhesion of soil particles. The main measurement methods include drying method, tensiometer method, resistance method and neutron method. The drying method can directly measure the soil moisture. The drying method was used to measure the soil moisture content. The calculation method is as follows:

(13) $$\omega =\frac{m_{ring+wet}-m_{ring+dry}}{m_{ring+wet}-m_{ring}}\times 100\%$$

In Formula (13), ω is soil moisture content, %. $m_{ring+wet}$ is the quality of cutting ring and soil sample before drying, g. $m_{ring+dry}$ is the quality of cutting ring and soil sample after drying, g. $m_{ring}$ is the quality of empty cutting ring, g.

(1.2) Soil density

Soil density is one of the important physical parameters of soil. The soil samples obtained by five-point sampling method were obtained according to the following Formula (14).

(14) $$\rho =\frac{m_{ring+soil}-m_{ring}}{v_{ring}}$$

In the Formula (14), ρ is soil density, g/cm^3^; $m_{ring+soil}$ is the quality of cutting ring and soil, g; $m_{ring}$ is the quality of cutting ring, g; and $v_{ring}$ is the volume of cutting ring, cm^3^.

(1.3) Soil Poisson’s ratio

Soil Poisson ‘s ratio refers to the ratio of the absolute value of the transverse normal strain to the axial normal strain when the soil is under uniaxial compression. A texture analyzer was used to apply unidirectional pressure to the soil samples for compression deformation test, which was calculated according to the following Formulas (15)–(17) according to the definition of soil Poisson’s ratio:

(15) $$\mu =\left|\frac{\varepsilon_{x}}{\varepsilon_{y}}\right|=\frac{\frac{\Delta x}{x_{0}}}{\frac{\Delta y}{y_{0}}}$$

(16) $$\Delta x=\left|x_{1}-x_{0}\right|$$

(17) $$\Delta y=\left|y_{1}-y_{0}\right|$$

In the formulas, μ is soil Poisson’s ratio. $\varepsilon_{x}$ is transverse strain. $\varepsilon_{y}$ is axial strain. Δx is absolute transverse deformation, mm. Δy is absolute axial deformation, mm. $x_{0}$ is soil sample diameter before compression, mm. $x_{1}$ is soil sample diameter after compression, mm. $y_{0}$ is soil sample height before compression, mm. $y_{1}$ is soil sample height after compression, mm.

(1.4) Soil elastic modulus

Soil elastic modulus is the instantaneous compressive stress–strain modulus of soil sample, reflecting its resistance to elastic deformation. A texture analyzer was used to apply pressure to the soil sample at a fixed loading speed, the data of the force (F)-axial displacement (Δy) curve were obtained, and the elastic modulus of the soil sample was calculated according to the following Formulas (18) and (19):

(18) $$E=\frac{\left(\frac{F}{A}\right)}{\varepsilon }$$

(19) $$\varepsilon =\frac{\Delta y}{y_{0}}$$

In the formulas, E is soil elastic modulus, MPa. *F* is axial load applied to soil samples, N. A is contact area, mm^2^. ε is transverse strain of soil sample. Δy is the variation in soil sample height after compression, mm. $y_{0}$ is soil sample height before compression, mm.

Soil intrinsic parameters were obtained based on the above research methods, as shown in Table 2.

(2) Material contact and contact model parameters

Interaction parameters between soil and rubber were required for simulation test from references [21,22]. The recovery coefficient of soil–rubber was 0.61, the static friction coefficient of soil–rubber was 0.48, and the rolling friction coefficient of soil–rubber was 0.23.

(2.1) Physical test of repose angle

Soil repose angle is used to characterize the flow and friction of soil particles, and the repose angle test is often used as a discrete element parameter calibration of soil particles. Natural rest test method was used to calibrate soil particle parameters. The soil accumulation angle test device comprises funnel, bracket, base, etc.

**Figure 7:**
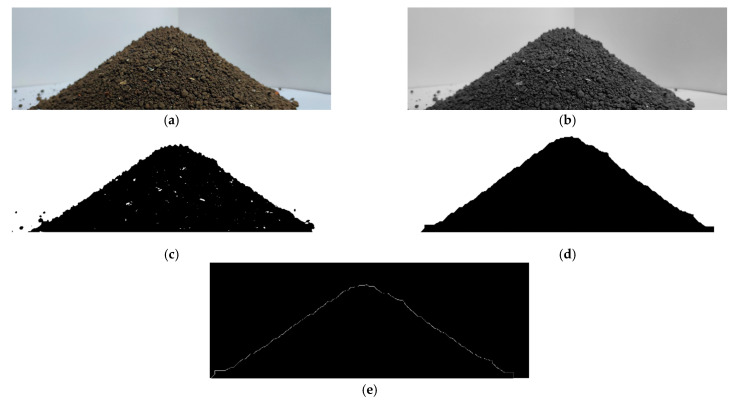
Soil repose angle processing flow chart of original images: (**a**) original image; (**b**) grayscale image; (**c**) binary image; (**d**) image after morphological processing; and (**e**) edge contour curve image.

After the soil was static without flow, and the slope was stable, the camera was used to obtain the static soil map, as shown in Figure 7a, and the image processing method of Matlab was used to process the static soil map and obtain the soil repose angle.

First, the image was grayed, as shown in Figure 7b. Based on the adaptive threshold segmentation, the image was binarized, as shown in Figure 7c. And, then, the morphological method was used to corrode and expand the image, as shown in Figure 7d. The edge contour curve of the soil accumulation state was extracted in Figure 7e, and the coordinates were read. Finally, the linear fitting tool was used to fit the coordinate data, and the soil repose angle was calculated from the slope [18]. The average soil repose angle obtained by multiple tests was 32.37°.

(2.2) Construction of virtual geometric model of repose angle test and setting of initial parameters

**Figure 8:**
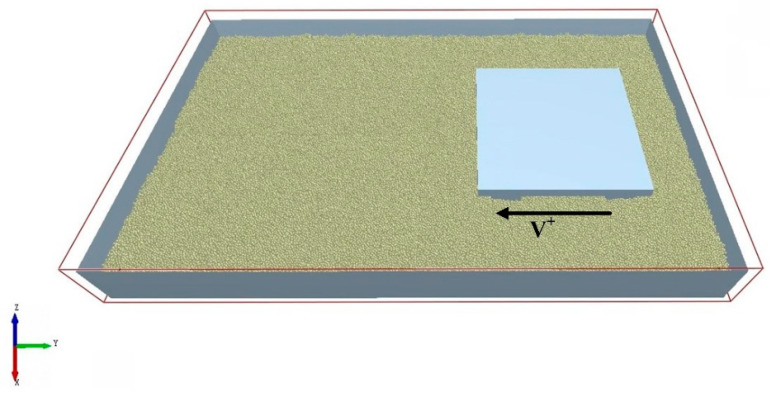
Track monomer–soil interaction simulation system.

Generic EDEM (Version 2020; DEM Solutions Ltd., Altair Engineering Inc., Troy, MI, USA, 2020). Material model database (GEMM) includes a variety of representative granular materials. The reference range of JKR surface energy, recovery coefficient, static friction coefficient and rolling friction coefficient can be obtained by inputting the simulation scale, soil density and repose angle into the database. Due to the small particle size of the soil particles, the soil particles were set as single spherical particles with a radius of 2 mm after considering the simulation results and the run time. According to the determined simulation parameters, firstly, 300 mm × 500 mm × 100 mm particle block was generated, particle factory generated 150,000 particles; then, the particle block was combined to generate 600 mm × 1000 mm × 100 mm particle bed with a total of 600,000 particles, and the constructed crawler monomer–soil interaction simulation system is shown in Figure 8.

The reference values of soil contact parameters and contact model parameters were as follows: JKR surface energy was 0–16 J/m^2^, recovery coefficient was 0.15–0.75, static friction coefficient was 0.2–1.16, and rolling friction coefficient was 0–0.2. Then, the Box–Behnken optimization method of Design-Expert 13 software was used to design parameter calibration experiments of 29 test points with 4 factors and 3 levels. The test factors were JKR surface energy, recovery coefficient, static friction coefficient and rolling friction coefficient, respectively. The simulation test factors and level settings are shown in Table 3.

The parameter calibration experiment index was simulated by soil repose angle, and the soil accumulation under different contact parameters was simulated by EDEM software Version 2020; Altair Engineering Inc., Troy, MI, USA. After the particles were stationary, the Clipping plane was added to the center of the funnel in the X and Y directions. The Protractor tool was used to obtain the soil repose angles of the two planes, and the average value was obtained as the calibration experiment index. Calibration experiment factor combination and soil repose angle simulation test results are shown in Table 4.

Regression fitting analysis was used to study the test data in the above table, and the soil repose angle Y regression model was obtained:

(20) $$\begin{array}{cc} Y=33.74+11.11A & +0.55B+1.40C+0.91D+0.21AB-2.67AC-1.90AD\\ & -1.42BC+0.88BD+0.16CD-0.65A^{2}+2.40B^{2}+2.32C^{2}\\ & +0.63D^{2} \end{array}$$

The decision coefficient (*R*^2^) was 0.9686 and corrected decision coefficient (R_adj_^2^) was 0.9373. It showed that the equation had high fitting degree and could replace the result analysis of real test points. The coefficient of variation (*C.V.*) was 5.47%, and signal-to-noise ratio (SNR) was 19.9382. It showed that the model has high reliability and accuracy. The variance analysis of the regression model obtained the analysis results shown in Table 5.

As can be seen from Table 5, *p* < 0.0001 < 0.01 indicated that this model was very significant and could be used to predict soil repose angle. Within the range of reference values of the given parameters, *A* had a very significant effect on the soil repose angle, *C* had a significant effect, but *B* and *D* had no significant effect. Among the first interaction terms of the four test factors, *AC* had significant effects on soil repose angle, while the other factors were not significant. Among the four quadratic test factors, *B*^2^ and *C*^2^ had significant effects on the repose angle, while the others were not significant. The regression model after removing insignificant factors was as follows:

(21) $$Y=33.74+11.11A+1.40C-2.67AC+2.40B^{2}+2.32C^{2}+0.63D^{2}$$

The optimization function of Design-Expert 13 software (Version 13; Stat-Ease, Inc., Minneapolis, MN, USA, 2022) was used to obtain the optimal solution of the experiment factors. JKR surface energy was 3.55 J/m^2^, soil–soil recovery coefficient was 0.41, soil–soil static friction coefficient was 0.53, and soil–soil rolling friction coefficient was 0.05. After many tests, the average repose angle of soil under the test conditions was 31.91°, and the relative error between the measured soil repose angle and the actual measurement was 1.42%, which met the allowable error range.

Due to the small size of soil particles, after considering the simulation results and running time, the soil particles were set as single spherical particles with a radius of 2 mm. According to the determined simulation parameters, the 300 mm × 500 mm × 100 mm particle block was generated first, and the particle factory generated 150,000 particles. Then, the particle blocks were combined to generate a 600 mm × 1000 mm × 100 mm particle bed, with a total of 600,000 particles.

### 2.4. Preparation of Rubber Track Monomer Sample

**Figure 9:**
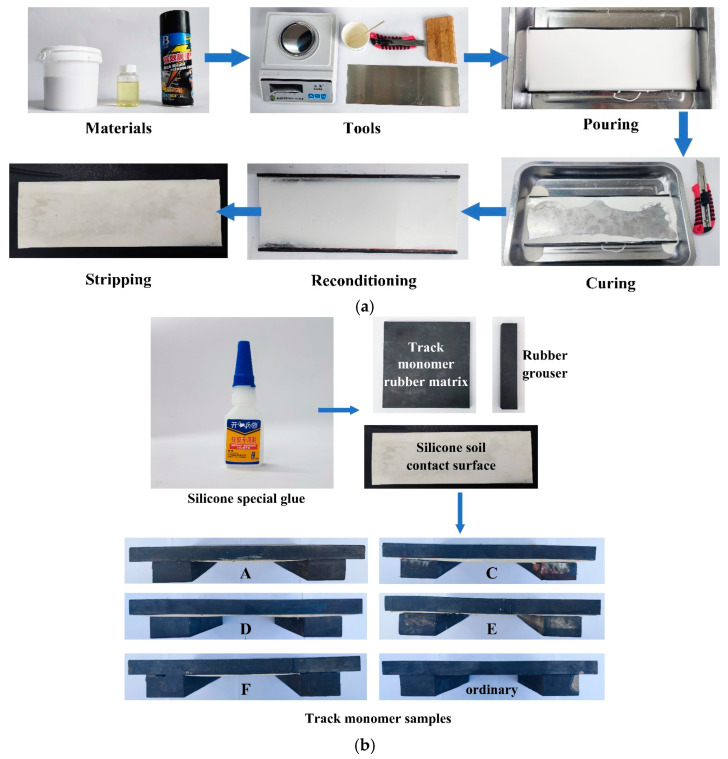
(**a**) Preparation process of silica gel contact surfaces. (**b**) Preparation process of track monomer samples.

Natural rubber plate (Shore’s hardness: 70A) was used as the monomer matrix of the track. The structural parameters of the selected natural rubber plate were as follows: length × width × height = 300 mm × 300 mm × 20 mm. The structure parameters were the same as those of the simulated track monomer model. All the one-line grousers were made of natural rubber material (Shore’s hardness: 70A), and the structural parameters were as follows: length × width × height = 300 mm × 50 mm × 30 mm. The structure parameters are the same as those of the simulated track monomer. Since the bionic structure of the track monomer matrix was unconventional, silicone material with Shore’s hardness of 70A was selected considering the material properties. The bionic structure of the track monomer matrix contact surface was prepared by the mold preparation method. The specific production process was as follows [22], as shown in Figure 9a:

(1) Mold preparation: According to the designed three-dimensional model of the bionic structure of the track monomer, the silica gel pouring mold was designed and manufactured by laser wire cutting technology.
(2) Colloid preparation: In order to increase the fluidity of silica gel material, silica gel material and curing agent were added in the container at a ratio of 100:1.8 and stirred in the same direction to break the large bubbles and reduce the bubbles.
(3) Pouring and curing: In order to facilitate the release and reduce the damage to the silicone during the release process, the release agent was evenly sprayed at the position 20–25 cm away from the mold during operation. The stirred silica gel material was slowly injected into the mold, while shaking the mold from side to side to ensure that the silica gel was fully injected. After injection into the mold, an area of stainless steel plate was placed on the surface, and release agent was evenly sprayed on the surface of the stainless steel plate in contact with the silicone material. Then, heavy objects were evenly placed on the surface of the stainless steel plate, and the mold was frozen in the refrigerator for 30 min to reduce bubbles. After the completion of pouring, in order to ensure the curing quality of the silicone, the mold was fixed for 4 h, the edge was trimmed, and the mold was removed.

Finally, according to the corresponding structural parameters, the track monomer rubber matrix, the silicone soil contact surface and the rubber grousers were spliced and bonded to form the track monomer samples, as shown in Figure 9b.

### 2.5. Building of Soil Tank Test System

**Figure 10:**
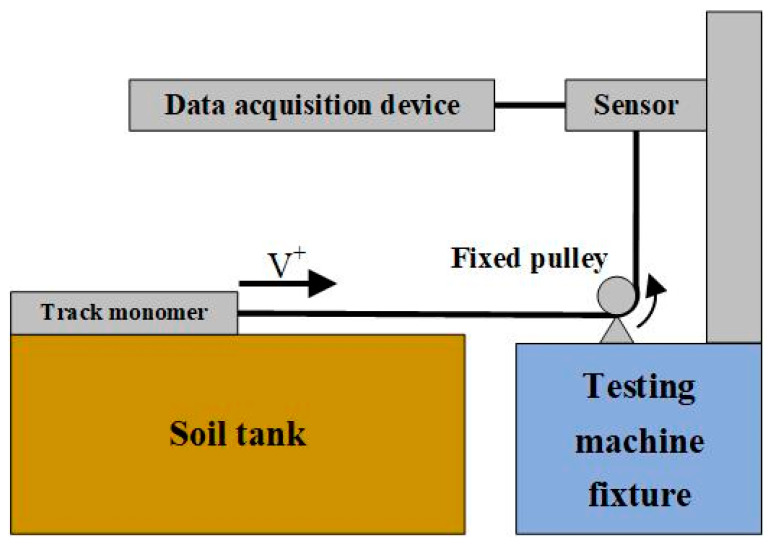
Schematic diagram of soil tank test system.

The research using the soil tank test system mainly includes the soil tank test bench (length × width × height = 1000 mm × 600 mm × 700 mm), tensile strength testing machine (XL-969S-500, Guangdong Xianglong Test Equipment Co., LTD., Guangdong, China) and data acquisition device laptop (Intel(R) Core(TM) i5-6300HQ CPU @ 2.3 GHz processor), etc., The soil tank test system [22] is shown in Figure 10.

**Figure 11:**
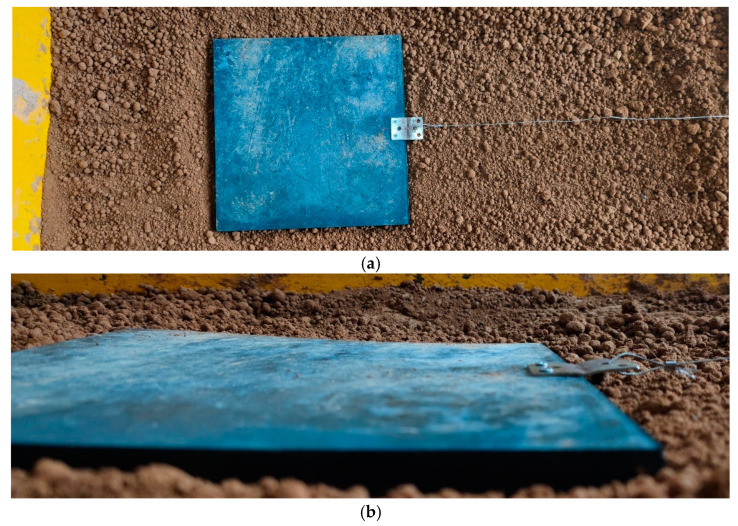
Layout diagram of track monomer sample: (**a**) Placement diagram of track monomer sample. (**b**) Settlement diagram of track monomer sample.

The track monomer traction test process was as follows:

(1) First, the soil loosening tool was used to sort out the soil, and the track monomer was smoothly placed on the soil surface according to the length of the traction wire rope. When placed, the geometric center line of the track monomer was in the same straight line with the fixed pulley. At the same time, in order to avoid the boundary effect of soil disturbed by the track monomer touching the inner wall of the soil tank, it was necessary to ensure that the boundary of the track monomer was more than 10 cm away from the inner wall of the soil tank when placing the track monomer.
(2) After the track monomer was placed according to the requirements, the track monomer was sunk into the soil to ensure that the soil was not over the grouser, the silica gel contact surface of the monomer sample completely contacted the soil, and, then, the test began. The placement of track monomer sample is shown in Figure 11.

(3) The traction process should ensure that the traction position of the testing machine and the fixed pulley were in the same straight line. During the test, the traction speed was set to 480 mm/min, which was the same as the horizontal motion speed of the track monomer in the simulation test (the second stage motion speed). The test was stopped when the traction distance was set to 200 mm, and the traction distance was the same as the horizontal movement distance of the simulation test (the second stage movement distance). The test data were derived, and the next set of tests were carried out.

The above operation process was repeated for each track monomer sample test, and the data accuracy was ensured by multiple tests. The test data were summarized and analyzed.

## 3. Results

### 3.1. Analysis of Discrete Element Simulation Results

**Figure 12:**
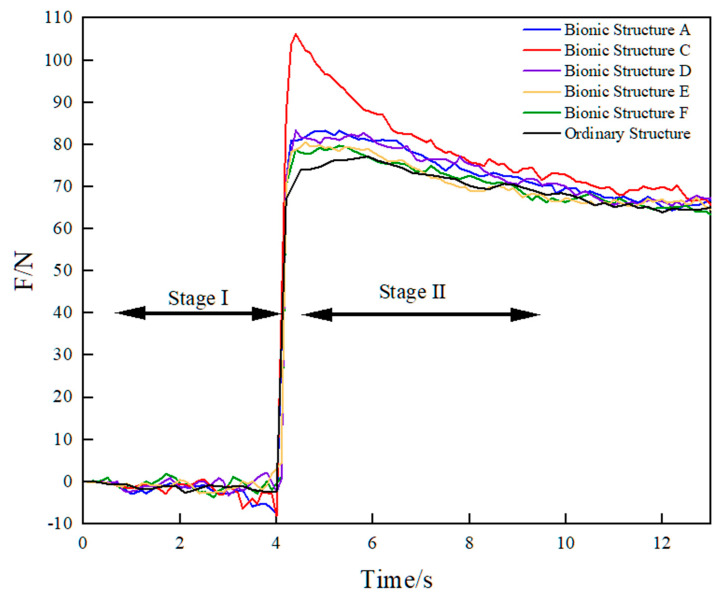
Simulation force changes in six track monomer structures.

In the simulation system of track monomer–soil interaction, the bionic structure and the ordinary structure of the track monomer were simulated, and the adhesion properties of different monomer structures were compared and analyzed. In the simulation, the movement direction of the track monomer structure was considered according to the spine structure and movement direction of the goat. After importing the track monomer structure model, it was set to have two-stage motion. The movement in the first stage was vertical downward movement with a speed of 8 mm/s until the spine was completely submerged into the soil particles. The initial position of the track monomer in this stage should ensure that no part of the track had contact with the particle bed. The second stage of motion was a horizontal linear motion with a speed of 8 mm/s. The direction of motion was shown in Figure 12, and the distance of motion was 200 mm. When moving, the soil particles in front of the track monomer structure should not exceed the simulation calculation domain to avoid the boundary effect affecting the simulation results.

(1) Numerical analysis of tractive force

The software derived the resultant force in the *Y*-axis direction of the model, and the force of the designed bionic structure A, C, D, E, F and the ordinary structure of the track monomer were drawn in the same figure, as shown in Figure 12.

As shown in Figure 12, although there were force fluctuations, the overall trend of force changes in the six structures was the same. When the track monomer moved vertically in the first stage and horizontally in the second stage, the forces of the six structures all increased sharply in a short time, then gradually decreased, and the curves tended to be stable. The maximum adhesion value (that is, the maximum force exerted by the track monomer) of the six track monomer structures without skid was collected, as shown in Table 6. It was found that the adhesion performance of the bionic structure was superior to that of the ordinary structure, indicating that the addition of bionic curve on the soil contact surface of the track monomer matrix could significantly improve the adhesion performance. And the bionic structure C had the best adhesion performance. Comparing the bionic structures A and F and the bionic structure C, D and E, it was found that the adhesion performance of the track monomer decreased with the increase in the number of layout bionic curves, and the adhesion performance of the track monomer with a single bionic curve was the best. Comparing the bionic structures A and C and the bionic structures D and F, it was found that the adhesion performance of the bionic structure C was the largest; that is, the bionic curve was arranged on the complete contact surface of the track monomer matrix to obtain the best adhesion performance.

(2) Contact force field of soil particles

The stress state of soil particles directly reflected the interaction between track monomer and soil, which could provide a basis for the study of the adhesion performance of track monomer. Therefore, in order to further explore the intrinsic adhesion mechanism of the track monomer bionic structure, the contact force field of soil particles was micro-analyzed based on the post-processing function of EDEM software. When the adhesion of the track monomer was maximum, the 400 mm × 400 mm × 80 mm area was selected with the track monomer as the center to cover the soil particles accumulated in the positive direction of the track monomer and the soil particles acted on by the grouser.

**Figure 13:**
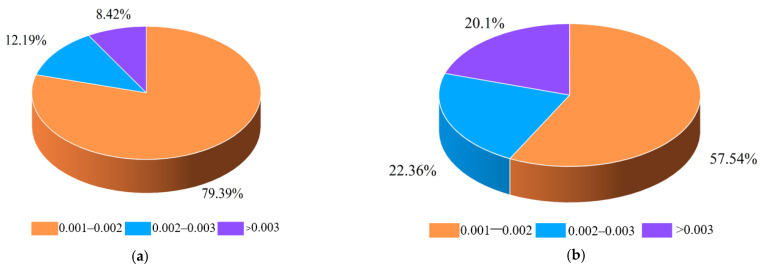

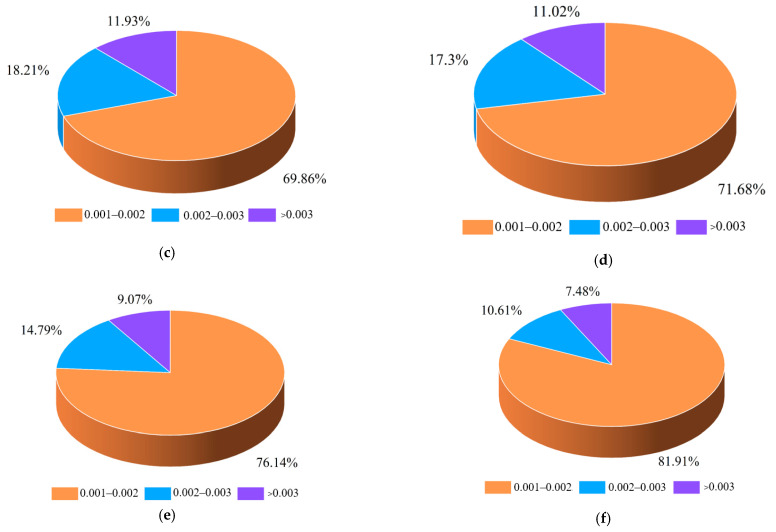
Proportional distribution of stressed particles of track monomer structures: (**a**) Particle proportion distribution of bionic structure A. (**b**) Particle proportion distribution of bionic structure C. (**c**) Particle proportion distribution of bionic structure D. (**d**) Particle proportion distribution of bionic structure E. (**e**) Particle proportion distribution of bionic structure F. (**f**) Particle proportion distribution of ordinary structure.

The proportional distribution of stressed particles of track monomer structures is shown in Figure 13. Due to the energy transfer and attenuation between particles, only soil particles with stress values greater than 0.001 N were counted in this study. The statistical analysis of the proportion distribution of stressed particles showed that the bionic structure C had the smallest proportion of particles, followed by bionic structure D. And the track monomeric had the largest proportion of ordinary structural particles in the range of 0.001–0.002 N and 0.002–0.003 N. On the contrary, in the maximum stress range, that is, greater than 0.003 N, the bionic structure C had the largest particle proportion, followed by bionic structure D, and the common structure had the smallest particle proportion. It corresponded to the conclusion that bionic structure C had the best optimization effect on adhesion performance, and bionic structure D had the second optimization effect. And the adhesion performance of the bionic structure was better than that of the ordinary structure.

### 3.2. Analysis of Soil Tank Verification Test Results

**Figure 14:**
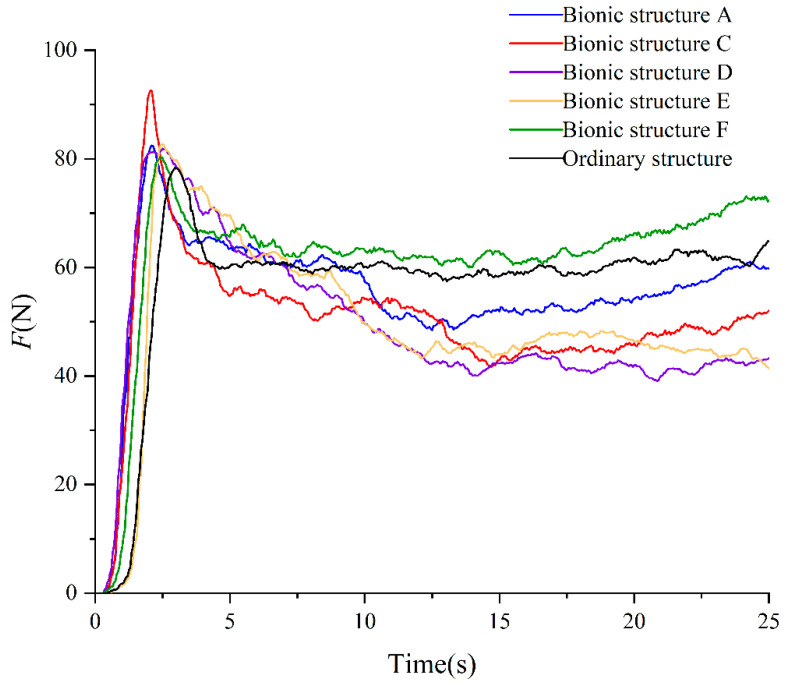
Traction–time curves of six track monomer samples.

The actual soil environment was complex, which was different from the simulation system of track mono-soil interaction based on EDEM discrete element software. In order to verify the reliability of the simulation results of the track monomer structure, the self-made soil tank test system was used to complete the traction test of the track monomer, and the test results were analyzed to obtain the bionic structure with the best adhesion performance. The single traction–time curves of track monomer samples are shown in Figure 14.

According to the analysis in Figure 14, the curve trend of the soil tank test results of six kinds of track monomer samples was the same, and the curve trend was consistent with the simulation test results. Due to the different pretension degree of traction wire rope, the time of sudden increase in the traction curve was different. When the track monomer began to shear the soil, the tractive force increased to the maximum value in a short time. It was considered that the maximum tractive force in the effective time of the test was the adhesion force of the track monomer. Then, as the track moved, the traction gradually decreased, and soil accumulation began to occur in the front of the track monomer. Due to the soil fluidity, the volume of accumulated soil was basically unchanged. And the influence on the track monomer sample was basically unchanged, so the tractive force curve of the monomer sample was basically stable. Because the real test environment was complicated, the curve fluctuation was obvious. The average adhesion value of the five groups of tests was selected as the adhesion value of the track monomer sample. The simulation results of track monomer structures and soil tank test are shown in Table 7.

According to the analysis of Table 7, comparing monomer structure A and C and monomer structure D and F; that is, comparing the track monomer structure with the same curve arrangement and different curve placement, it could be found that the adhesion value of structures C and D was greater than that of structures A and F. It indicated that, compared with the monomer structure that only arranged a bionic curve on the partial contact surface (between the grousers), the monomer structure that arranged a curve on the full-contact surface had more obvious interaction with the soil, and the adhesion performance of the track monomer was better. It was because a larger soil disturbance area could be obtained when the curve was arranged on the full-contact surface, and the interaction between the track monomer and the soil was greater. By comparing the monomer structures A and F, and the monomer structures D, E and F; that is, comparing the monomer structure with a different curve arrangement and the same curve placement, it was found that the adhesion value of the track monomer increased with the decrease in the number of curve arrangements, and the track monomer with a single curve had the best adhesion performance. It was because the height of the structure of the contact surface increased with the decrease in the number of curves, resulting in the increase in the subsidence of the contact surface in the soil. According to the stress analysis of track monomer, the increase in subsidence would change the horizontal force on the soil contact surface of track monomer, thus improving the adhesion performance of track monomer.

Due to the complexity of the soil tank test environment, the maximum error of the tractive force of the track monomer obtained was 9.02% compared with the simulation result, which met the accuracy requirements and verifies the feasibility of the simulation analysis. The adhesion of the optimal bionic structure C was 19.22 N higher than that of the ordinary structure, which verified the superiority of the track monomer bionic structure design.

## 4. Conclusions

(1) The track monomer bionic structure was designed based on the above spinal space curve equation. Based on EDEM discrete element simulation parameter calibration and measurements, taking the maximum tractive force (adhesion) and the surface pressure of track monomer at the time of maximum adhesion as evaluation indexes, it was found that positive deformation of the bionic curve was the best, and the adhesion performance of track monomer with a positive deformation bionic curve was the best.
(2) In order to further analyze the influence of bionic curve arrangement and placement on the adhesion performance of the track monomer, different bionic structures of the track monomer were designed. Soil trench test results showed that the adhesion force was 19.22 N higher than that of the ordinary structure of the tracked monomer, and the maximum error between simulation analysis and test verification was 9.02%, which verified the superiority of the design of the bionic structure of the tracked monomer.

## Figures and Tables

**Table 1:** De-noising results of three-dimensional force in space.

Evaluation Indexes	The Three-Dimensional Force	Moving	Lowess	Loess	Sgolay	Rlowess	Rloess
SNR	$F_{x1}$	23.7108	24.9864	26.0318	25.3184	23.8431	25.3032
	$F_{x2}$	35.5948	37.5373	39.3775	38.2828	34.4383	37.8937
	$F_{y1}$	53.6476	56.2609	58.9847	57.1225	52.0155	56.7734
	$F_{y2}$	54.4331	58.0150	62.0709	59.6972	50.8870	57.4044
	$F_{z1}$	59.6121	63.7341	66.8021	65.9467	55.5029	62.9150
	$F_{z2}$	62.3318	66.1048	68.7752	67.9366	56.2001	64.7932
RMSE	$F_{x1}$	0.0118	0.0102	0.0090	0.0098	0.0116	0.0098
	$F_{x2}$	0.0171	0.0137	0.0111	0.0125	0.0195	0.0131
	$F_{y1}$	0.0198	0.0147	0.0107	0.0133	0.0239	0.0138
	$F_{y2}$	0.0371	0.0246	0.0154	0.0203	0.0559	0.0264
	$F_{z1}$	0.0515	0.0320	0.0225	0.0248	0.0826	0.0352
	$F_{z2}$	0.0806	0.0522	0.0384	0.0423	0.1634	0.0607
*R* ^2^	$F_{x1}$	0.9672	0.9756	0.9808	0.9774	0.9682	0.9773
	$F_{x2}$	0.9842	0.9899	0.9934	0.9915	0.9794	0.9907
	$F_{y1}$	0.9954	0.9975	0.9986	0.9979	0.9932	0.9977
	$F_{y2}$	0.9943	0.9975	0.9990	0.9983	0.9871	0.9971
	$F_{z1}$	0.9983	0.9994	0.9997	0.9996	0.9957	0.9992
	$F_{z2}$	0.9989	0.9996	0.9998	0.9997	0.9956	0.9994

**Table 2:** Soil intrinsic parameters.

Soil Intrinsic Parameter	Value
moisture content	12.73%
density	1.50 g/cm^3^
Poisson’s ratio	0.31
elastic modulus	1.60 × 10^7^ Pa

**Table 3:** Simulation test factors and level design table.

Variable	Experimental Factors	Low Level	Center Level	High Level
A	JKR surface energy	0	8	16
B	recovery coefficient	0.15	0.45	0.75
C	static friction coefficient	0.20	0.68	1.16
D	rolling friction coefficient	0	0.1	0.2

**Table 4:** Calibration experiment factor combination and soil repose angle simulation test results.

No.	Experiment Factors				Experiment Index
	JKR Surface Energy (J/m^2^)	Recovery Coefficient	Static Friction Coefficient	Rolling Friction Coefficient	Soil Repose Angle (°)
1	0	0.15	0.68	0.1	24.17
2	0	0.45	0.2	0.1	20.34
3	0	0.45	0.68	0	20.24
4	0	0.45	0.68	0.2	26.12
5	0	0.45	1.16	0.1	29.00
6	0	0.75	0.68	0.1	22.73
7	8	0.45	0.68	0.1	34.20
8	8	0.15	0.2	0.1	33.84
9	8	0.15	0.68	0	36.97
10	8	0.15	0.68	0.2	37.34
11	8	0.15	1.16	0.1	40.90
12	8	0.45	0.2	0	34.34
13	8	0.45	0.2	0.2	35.25
14	8	0.45	0.68	0.1	33.33
15	8	0.45	0.68	0.1	33.73
16	8	0.45	0.68	0.1	33.75
17	8	0.45	0.68	0.1	33.69
18	8	0.45	1.16	0	34.86
19	8	0.45	1.16	0.2	36.41
20	8	0.75	0.2	0.1	41.00
21	8	0.75	0.68	0	35.25
22	8	0.75	0.68	0.2	39.12
23	8	0.75	1.16	0.1	42.38
24	16	0.15	0.68	0.1	44.89
25	16	0.45	0.2	0.1	47.96
26	16	0.45	0.68	0	47.29
27	16	0.45	0.68	0.2	45.57
28	16	0.45	1.16	0.1	45.95
29	16	0.75	0.68	0.1	44.27

**Table 5:** Regression model variance analysis results.

Source of Variance	Quadratic Sum	Degree of Freedom	Mean Square	*F* Value	*p* Value	Significance
model	1646.74	14	117.62	30.89	<0.0001	**
*A*	1481.41	1	1481.41	389.07	<0.0001	**
*B*	3.67	1	3.67	0.9650	0.3426	
*C*	23.44	1	23.44	6.16	0.0264	*
*D*	9.83	1	9.83	2.58	0.1304	
*AB*	0.1681	1	0.1681	0.0441	0.8366	
*AC*	28.46	1	28.46	7.48	0.0161	*
*AD*	14.44	1	14.44	3.79	0.0718	
*BC*	8.07	1	8.07	2.12	0.1676	
*BD*	3.06	1	3.06	0.8043	0.3850	
*CD*	0.1024	1	0.1024	0.0269	0.8721	
*A* ^2^	2.70	1	2.70	0.7087	0.4140	
*B* ^2^	37.25	1	37.25	9.78	0.0074	**
*C* ^2^	34.84	1	34.84	9.15	0.0091	**
*D* ^2^	2.61	1	2.61	0.6842	0.4220	
pure error	0.3824	4	0.0956			
total	1700.05	28				

Note: “p≤0.01” means that the item is very significant (**), “0.01<p≤0.05” means that the item is significant (*), and “p>0.05” indicates that the item is not significant.

**Table 6:** The maximum adhesion values of track monomer structures.

No.	Structure	Maximum Adhesion Value (N)	Adhesion Value Optimization Effect (N)
1	A	83.29	6.13
2	C	106.27	29.11
3	D	83.35	6.19
4	E	80.54	3.38
5	F	79.76	2.60
6	Ordinary	77.16	--

**Table 7:** Simulation and test results of track monomer structures.

No.	Structures	Simulated Adhesion Value (N)	Test Adhesion Value (N)	Error (%)
1	A	83.29	85.04	2.06
2	C	106.27	97.48	9.02
3	D	83.35	86.41	3.54
4	E	80.54	82.77	2.69
5	F	79.76	80.39	0.78
6	Ordinary	77.16	78.26	1.41

## Data Availability

The original contributions presented in this study are included in the article.
